# CB1 enhanced the osteo/dentinogenic differentiation ability of periodontal ligament stem cells via p38 MAPK and JNK in an inflammatory environment

**DOI:** 10.1111/cpr.12691

**Published:** 2019-10-10

**Authors:** Wanhao Yan, Yangyang Cao, Haoqing Yang, Nannan Han, Xinling Zhu, Zhipeng Fan, Juan Du, Fengqiu Zhang

**Affiliations:** ^1^ Department of Periodontology Capital Medical University School of Stomatology Beijing China; ^2^ Laboratory of Molecular Signaling and Stem Cells Therapy Beijing Key Laboratory of Tooth Regeneration and Function Reconstruction Capital Medical University School of Stomatology Beijing China

**Keywords:** CB1, inflammation, MAPK signalling pathway, osteo/dentinogenic differentiation, periodontal ligament stem cells (PDLSCs)

## Abstract

**Objectives:**

Periodontitis is an inflammatory immune disease that causes periodontal tissue loss. Inflammatory immunity and bone metabolism are closely related to periodontitis. The cannabinoid receptor I (CB1) is an important constituent of the endocannabinoid system and participates in bone metabolism and inflammation tissue healing. It is unclear whether CB1 affects the mesenchymal stem cell (MSC) function involved in periodontal tissue regeneration. In this study, we revealed the role and mechanism of CB1 in the osteo/dentinogenic differentiation of periodontal ligament stem cells (PDLSCs) in an inflammatory environment.

**Materials and methods:**

Alkaline phosphatase (ALP) activity, Alizarin Red staining, quantitative calcium analysis and osteo/dentinogenic markers were used to assess osteo/dentinogenic differentiation. Real‐time RT‐PCR and Western blotting were employed to detect gene expression.

**Results:**

CB1 overexpression or CB1 agonist (10 µM R‐1 Meth) promoted the osteo/dentinogenic differentiation of PDLSCs. Deletion of CB1 or the application of CB1 antagonist (10 µM AM251) repressed the osteo/dentinogenic differentiation of PDLSCs. The activation of CB1 enhanced the TNF‐α‐ and INF‐γ‐impaired osteo/dentinogenic differentiation potential in PDLSCs. Moreover, CB1 activated p38 MAPK and JNK signalling and repressed PPAR‐γ and Erk1/2 signalling. Inhibition of JNK signalling could block CB1‐activated JNK and p38 MAPK signalling, while CB1 could activate p38 MAPK and JNK signalling, which was inhibited by TNF‐α and INF‐γ stimulation.

**Conclusions:**

CB1 was able to enhance the osteo/dentinogenic differentiation ability of PDLSCs via p38 MAPK and JNK signalling in an inflammatory environment, which might be a potential target for periodontitis treatment.

## 1 INTRODUCTION

Periodontitis is a chronic inflammatory disease caused by bacteria and various other factors and can lead to alveolar bone resorption, tooth movability and migration, and ultimately lead to tooth loss.^1^, ^2^, ^3^ At present, traditional periodontal treatment methods cannot achieve an ideal regeneration of periodontal tissue loss.^4^ Therefore, the essential question of periodontal therapy is to reconstruct the lost periodontal tissues. Mesenchymal stem cells (MSCs) are reported to be excellent seed cells for regeneration treatments due to their multidirectional differentiation capability. In‐depth studies on the utility of MSCs in periodontal tissue regeneration have reported that using MSCs as seed cells implanted into periodontal lesion areas via cell sheet or local injection can enhance periodontal tissue regeneration.^5^, ^6^, ^7^, ^8^ Periodontal ligament stem cells (PDLSCs) are a type of tissue‐specific MSC and behave according to the prevailing MSC speciality, such as in the expression of key markers, self‐renewal, multipotency, and immune suppression responses, and they can differentiate into mesenchymal cell lineages in vitro and in vivo.^4^, ^9^, ^10^, ^11^ However, several key PDLSC surface markers, including STRO‐1, CD90, OCT‐4 and CD146, are significantly abnormally expressed in inflamed tissues compared with healthy PDLSCs. Meanwhile, the inflammatory environment also weakens the directional differentiation function of PDLSCs, and it is difficult for these damaged PDLSCs to achieve the requirements of periodontal injury tissue regeneration.^12^, ^13^ Thus, enhancing PDLSC function in inflammatory environments is a key issue for defective periodontal regeneration in periodontitis.

Studies have found that the cell membrane receptor‐ligand pathway is an important mechanism of alveolar bone loss in periodontitis.^14^ Activation of the receptor‐ligand pathway recruits MSCs, and the activated MSCs act as a pivotal factor in periodontitis alveolar bone resorption.^14^, ^15^ Studies have reported that better periodontal regeneration was obtained by improving seed cell function through the addition of cytokines.^8^, ^16^ These studies suggested that the damaged receptors on the MSC membrane surface in periodontitis could be reactivated via genetic modification or specific activators. Cannabinoid receptor I (CB1) is an important part of the endogenous cannabinoid system (ECS); it is a G protein‐coupled receptor with seven transmembrane domains.^17^ CB1 can transmit signals via the membrane receptor‐ligand pathway.^18^ The expression of CB1 results in a decrease in inflamed periodontal ligament (PDL) tissues.^19^ The endogenous cannabinoid anandamide (AEA) and the CB1 and cannabinoid receptor II (CB2) are expressed in periodontal tissues and participate in the physiological protection from ultra‐inflammatory responses though mediating anti‐inflammatory‐related cellular pathways.^20^ It has been reported that lifetime treatment with the CB1‐specific agonist meth‐AEA (an AEA analogue) significantly attenuates the loss of alveolar bone in a rat periodontitis model.^21^ Previous studies have found that CB1 is activated during the osteogenic differentiation of bone marrow mesenchymal stem cells (BMSCs) in mice, and CB1 enhances the early osteogenic ability of BMSCs and influences their survival during acute stress.^22^, ^23^ One study has reported that CB1 regulates BMSC migration/homing in mice, and this mediated BMSC function can be enhanced by the CB1‐specific agonists ACEA and weakened by the CB1‐specific antagonist AM281.^24^ Studies also have reported that CB1 participates in bone regulation and bone metabolism in bone tissue.^25^, ^26^, ^27^, ^28^ CB1^−/−^ mice develop osteoporosis, which is further exacerbated with ageing as a result of reduced bone formation and the accumulation of fat cells.^29^ Furthermore, CB1 has a regulatory role in the healing of inflamed tissues.^20^, ^30^ CP55940, an agonist of CB1, promotes human gingival fibroblast (HGF)‐mediated wound healing by activating CB1.^30^

Regulatory mechanism investigations have reported that CB1 can activate the mitogen‐activated protein kinases (MAPK) signalling pathways, including the p38 mitogen‐activated protein kinase (p38 MAPK), c‐Jun N‐terminal kinase (JNK) and extracellular signal‐regulated protein kinases 1 and 2 (Erk1/2) pathways, which could further regulate cellular proliferation, survival and differentiation.^31^, ^32^ The MAPKs consist of a series of highly homologous protein kinases and are present in most cells; they help to transduce extracellular stimuli into cells and nuclei and are involved in the p38 MAPK, JNK and Erk1/2 signalling pathways.^33^, ^34^ The MAPK signalling pathways are necessary in human adipose‐derived stem cell (ADSC) osteogenesis.^35^ The p38 MAPK and JNK signalling pathways are activated in PDLSCs after treatment with low‐intensity pulsed ultrasound (LIPUS), and the p38 MAPK‐specific inhibitor SB203580 and the JNK‐specific inhibitor SP600125 can modulate LIPUS‐stimulated PDLSC proliferation.^36^ Erk1/2 is closely related to PDLSC proliferation, differentiation and cementum formation.^37^, ^38^ In addition, several congeners of the endocannabinoids also act within the wider receptor spectrum, including peroxisome proliferator‐activated receptors (PPARs) and other G protein‐coupled receptors (GPCRs). PPARs are ligand‐activated receptors in the nuclear hormone receptor family.^39^ In the inflammatory response, PPAR‐γ inhibits the production of inflammatory signalling pathways and inflammatory mediators.^40^ Moreover, PPAR‐γ can regulate BMSCs to differentiate into osteoblasts or adipocytes.^41^ BMSCs from CB1^‐/‐^ mice increase the expression of PPAR‐γ.^29^ A previous study showed that miR‐29a sufficiently suppressed the expression of CB1, which further restored PPAR‐γ signalling.^42^ The activation of PPAR‐γ, often in conjunction with the activation of CB1, could mediate the anti‐inflammatory, analgesic, metabolic, neuroprotective, antitumour and cardiovascular effects of cannabinoids.^43^ However, the function and underling mechanism of CB1 in PDLSCs in an inflammatory environment remains unclear along with its involvement in periodontal regeneration.

Through analysing the dynamic changes in the local inflammatory microenvironment sites of damaged tissues, previous studies have shown that this process is accompanied by changes in the type and quantity of inflammatory cytokines; for example, high concentrations of pro‐inflammatory cytokines predominate in the acute inflammatory phase, including TNF‐α and INF‐γ. Further studies have shown that high concentrations of inflammatory cytokines are sufficient to stimulate the immunosuppressive ability of MSCs, especially TNF‐α and INF‐γ, which are crucial in initiating the immunosuppressive effect of MSCs.^44^, ^45^ It can be seen that the MSC‐based therapeutic effect is impacted by the inflammatory conditions. In the current study, we used TNF‐α and INF‐γ to mimic an inflammatory environment, and we clarified the role and mechanism of CB1 in PDLSCs in this inflammatory environment. Our results showed that CB1 upregulated the osteo/dentinogenic differentiation of PDLSCs upon stimulation by the inflammatory factors TNF‐α and INF‐γ via activating the p38 MAPK and JNK signalling pathways.

## 2 MATERIALS AND METHODS

### 2.1 Cell cultures

As in our previous study, the PDLSCs used in the current study were isolated from impacted third molar human teeth that were obtained after informed patient agreement and following the rules approved by the Beijing Stomatological Hospital, Capital Medical University (Ethics Committee Agreement, Beijing Stomatological Hospital Ethics Review No. 2011‐02). These cells were cultivated and verified according to our previous protocol.^8^

In this study, 10 ng/mL tumour necrosis factor‐alpha (TNF‐α) (R&D Systems) and 100 ng/mL interferon‐gamma (INF‐γ) (R&D Systems) were used to stimulate the PDLSCs. Selective CB1 agonist, 10 µM R‐1 methanandamide (R‐1 Meth) and selective CB1 antagonist, 10 µM AM251 (Cayman Chemical) were used. The p38 MAPK‐specific inhibitor, 20 µM SB203580 (MedChemExpress), and the JNK‐specific inhibitor, 20 µM SP600125 (Merck), were used to stimulate the PDLSCs.

### 2.2 Plasmid construction and viral infection

The plasmid construction and viral infection procedures were carried out as in our previous study.^8^ The plasmids were constructed according to standard techniques, and all structures were verified by enzyme digestion and/or sequencing. Human full‐length CB1 cDNA that included an HA tag was constructed via a whole‐gene synthesis method. This HA‐CB1 sequence was inserted into the pQCXIN retroviral vector via the AgeI and BamH1 restriction sites. Viral packaging was prepared following the manufacturer's protocol (Clontech Laboratories). CB1 shRNA and control shRNA lentivirus were obtained from GenePharma. The targeted sequence for the control shRNA (Consh) was as follows: 5'‐TTCTCCGAACGTGTCACGTTTC‐3', and the targeted sequence for the CB1 shRNA (CB1sh) was as follows: 5’‐GCCGCAACGTGTTTCTGTTCA‐3’.

### 2.3 Reverse transcriptase‐polymerase chain reaction (RT‐PCR) and real‐time reverse transcriptase‐polymerase chain reaction (real‐time RT‐PCR)

RNA extraction, cDNA synthesis and real‐time RT‐PCR procedures were performed as previously described.^8^ Real‐time RT‐PCR reactions were performed according to the QuantiTect SYBR Green PCR kit (Qiagen) using an icycler iQ Multi‐colour Real‐time RT‐PCR Detection System. All primer sequences are stated in Table S1.

### 2.4 Alkaline phosphatase (ALP) activity assay and Alizarin red detection

PDLSCs were cultured in osteogenic‐inducing medium containing 100 μM/mL ascorbic acid, 2 mM β‐glycerophosphate, 1.8 mM KH_2_PO_4_ and 10 nM dexamethasone. The ALP activity was measured using an ALP activity kit (Sigma‐Aldrich) according to the manufacturer's protocol. After osteogenic induction of MSCs for 2 weeks, 70% ethanol and 2% Alizarin red (Sigma‐Aldrich) were used to fix and stain the cultured cells. The plates were then photographed and destained for 30 minutes at room temperature with 10% cetylpyridinium chloride. The absorbance of the cell cultures was measured at 562 nm on a multi‐plate reader, and the final calcium level was normalized according to the total protein concentration in duplicate plates.

### 2.5 Western blot analysis

The total protein extraction and the SDS‐polyacrylamide gel electrophoresis tests were performed as previously described.^8^ The primary antibodies used in this study were anti‐CB1 (Cat No. 93815; Cell Signalling Technology), anti‐CB2 (Cat No. ab3561; Abcam), mouse monoclonal anti‐HA (Clone No. C29F4; Cat No. MMS‐101P; Covance), anti‐phospho‐p38 MAPK (Cat No. 4631; Cell Signalling Technology), anti‐p38 MAPK (Cat No. 8690; Cell Signalling Technology), anti‐phospho‐JNK (Cat No. 4668; Cell Signalling Technology), anti‐JNK (Cat No. 9258; Cell Signalling Technology), anti‐phospho‐Erk1/2 (Cat No. 4377S; Cell Signalling Technology), anti‐Erk1/2 (Cat No. 4695S; Cell Signalling Technology) and anti‐PPAR‐γ (Cat No.59256; Abcam). The primary monoclonal antibodies for the housekeeping proteins were the monoclonal antibody against histone H3 (Cat No.10809; Santa Cruz Biotechnology) and β‐actin (Cat No. C1313; Applygen).

### 2.6 Statistical analysis

All statistical calculations were implemented by SPSS 10 statistical software (SPSS Inc). Student's *t* test or one‐way ANOVA was used to identify statistical significance, with *P* ≤ .05 considered to be significant.

## 3 RESULTS

### 3.1 Knock‐down of CB1 inhibited the osteo/dentinogenic differentiation of PDLSCs

**Figure 1:**
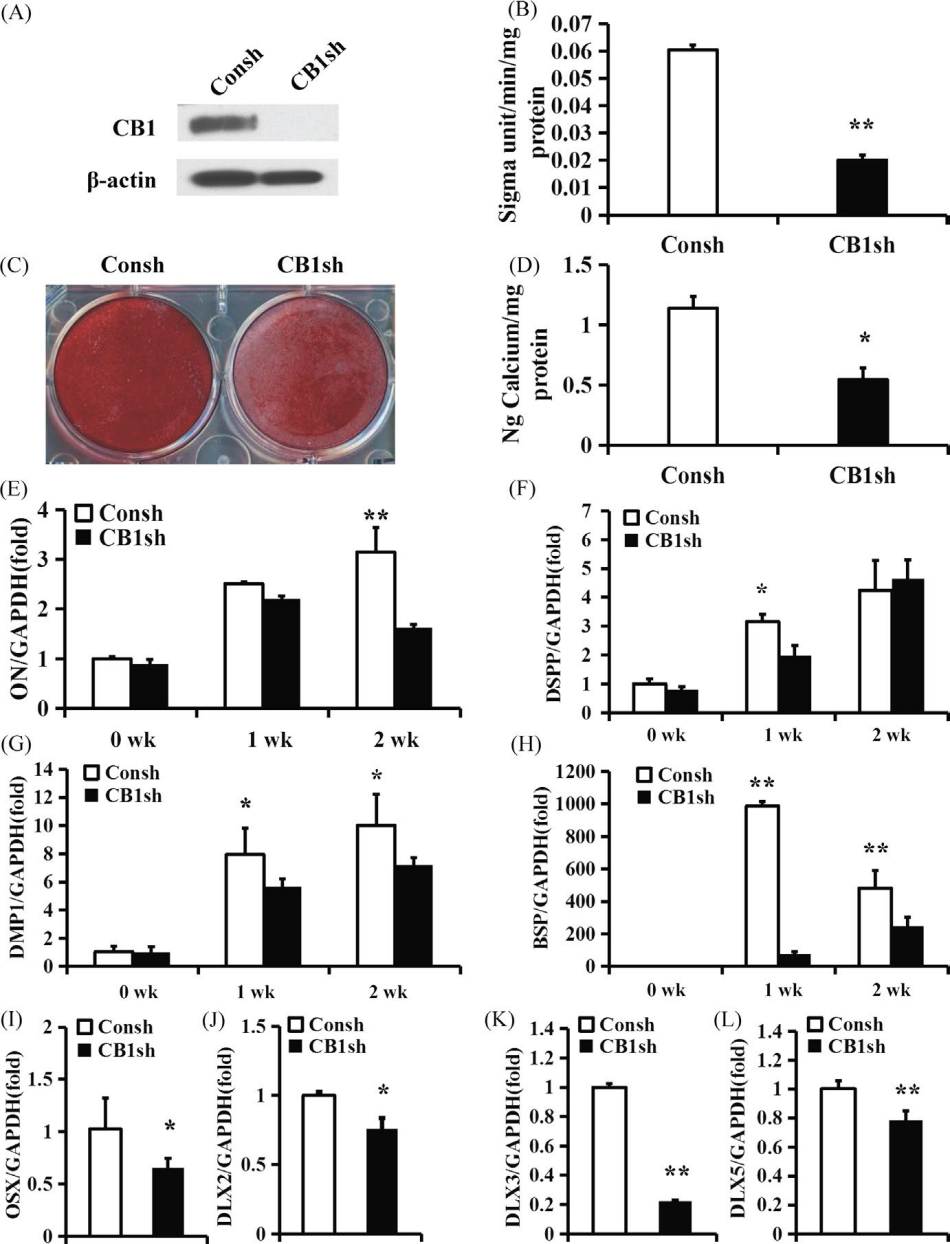
CB1 knock‐down inhibited the osteo/dentinogenic differentiation of PDLSCs. (A) Western blot results showed the knock‐down efficiency of CB1 shRNA in PDLSCs. β‐actin was used as an internal control. (B) ALP activity assay. (C) Alizarin Red staining. (D) Calcium quantitative analysis. (E‐H) Real‐time RT‐PCR results of the *ON* (E), *DSPP* (F), *DMP1* (G) and *BSP* (H) expression levels during PDLSC osteo/dentinogenic differentiation. (I‐L) Real‐time RT‐PCR results of *OSX* (I), *DLX2* (J), *DLX3* (K) and *DLX5* (L) expression levels in PDLSCs. GAPDH was used as an internal control. Student's *t* test was performed to determine statistical significance. Error bars represent the SD (n = 3). *P ≤ .05; **P ≤ .01

CB1 shRNA lentivirus was used to delete CB1 expression in PDLSCs; after 3 days of puromycin selection (1 µg/mL), the knock‐down efficiency was verified by Western blot (Figure 1A). After 5 days of culturing in osteogenic‐inducing medium, the ALP activity assay results indicated that CB1 knock‐down reduced PDLSC ALP activity compared with the control group (Consh group) (Figure 1B). Alizarin red staining and the calcium quantitative measurement results revealed that the deletion of CB1 inhibited in vitro mineralization of PDLSCs compared with the control group (Figure 1C,D). Compared with the control group, the real‐time RT‐PCR results in the CB1sh group showed that *ON* expression was significantly reduced at 2 weeks (Figure 1E), *DSPP* expression was significantly decreased at 1 week (Figure 1F) and the *DMP1* and *BSP* expression levels were significantly reduced at 1 and 2 weeks after osteogenic induction (Figure 1G,H). Furthermore, *OSX*, *DLX2*, *DLX3* and *DLX5* were also significantly reduced in the CB1sh group compared to the control group (Figure 1I‐L).

### 3.2 Activation of CB1 promoted the osteo/dentinogenic differentiation of PDLSCs

**Figure 2:**
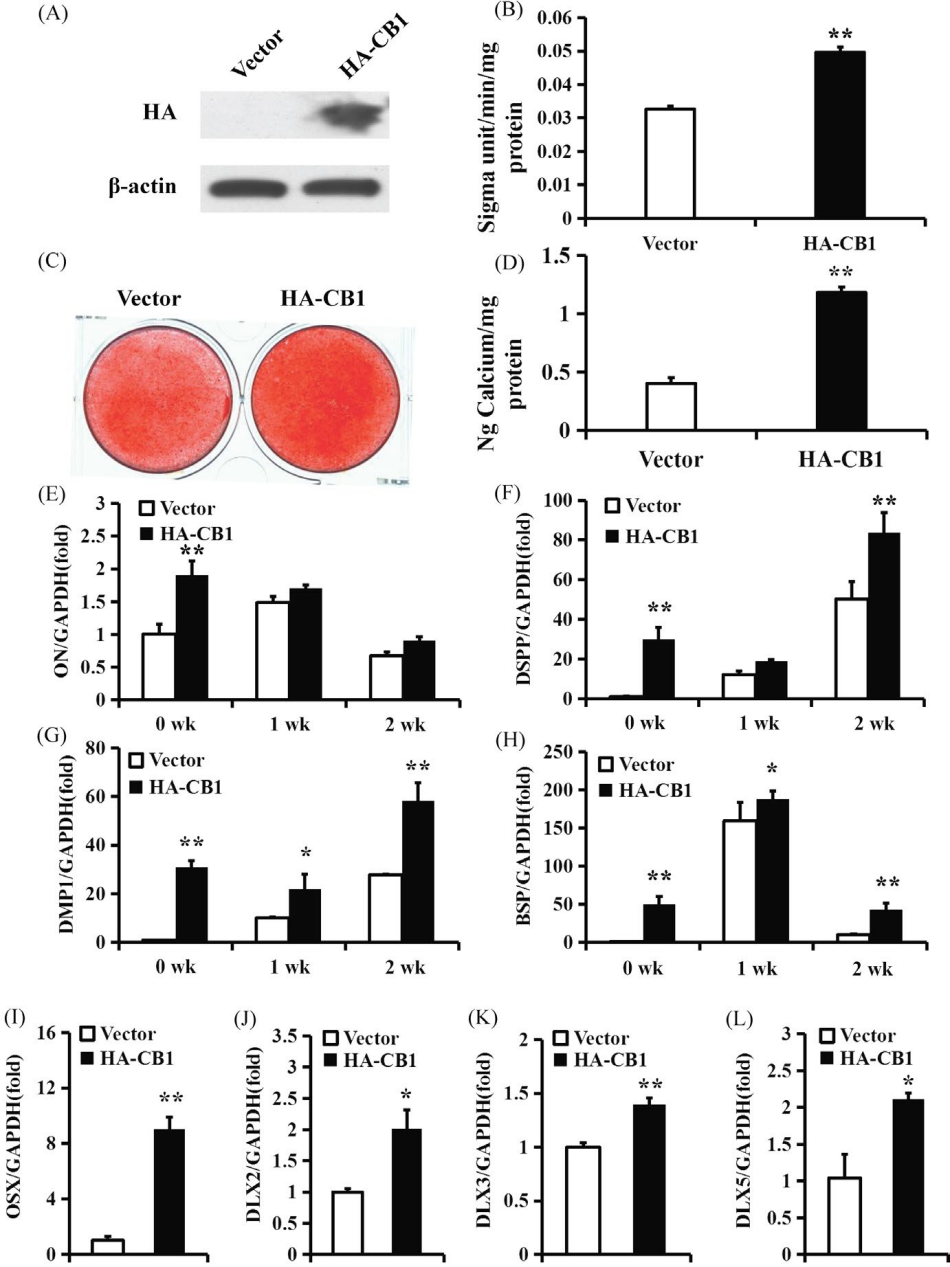
Overexpression of CB1 enhanced the osteo/dentinogenic differentiation of PDLSCs. (A) Western blot results showed the overexpression efficiency of HA‐CB1 in PDLSCs. β‐actin was used as an internal control. (B) ALP activity assay. (C) Alizarin Red staining. (D) Calcium quantitative analysis. (E‐H) Real‐time RT‐PCR results of the *ON* (E), *DSPP* (F), *DMP1* (G) and *BSP* (H) expression levels during PDLSC osteo/dentinogenic differentiation. (I‐L) Real‐time RT‐PCR results of *OSX* (I), *DLX2* (J), *DLX3* (K) and *DLX5* (L) expression levels in PDLSCs. GAPDH was used as an internal control. Student's *t* test was performed to determine statistical significance. Error bars represent the SD (n = 3). *P ≤ .05; **P ≤ .01

The HA‐CB1 sequence was constructed in a retroviral vector and then transduced into PDLSCs by retroviral infection. After selection of 600 µg/mL G418 for 10 days, the overexpression efficiency was confirmed by Western blot (Figure 2A). After 5 days of culturing in osteogenic‐inducing medium, the ALP activity indicated that the overexpression of CB1 enhanced the ALP activity of PDLSCs compared with the control group (Vector group) (Figure 2B). Alizarin red staining and quantitative calcium measurements revealed that the overexpression of CB1 promoted the in vitro mineralization of PDLSCs compared with the control group (Figure 2C,D). The real‐time RT‐PCR results showed that *ON* expression was significantly increased at 0 weeks (Figure 2E), the *DSPP* expression was significantly increased at 0 and 2 weeks (Figure 2F) and the *DMP1* and *BSP* expression levels were significantly increased at 0, 1 and 2 weeks after osteogenic induction in CB1 overexpressed PDLSCs compared with the control group (Figure 2G,H). Furthermore, the *OSX*, *DLX2*, *DLX3* and *DLX5* expression levels were increased in CB1 overexpressing PDLSCs compared to the control group (Figure 2I‐L).

**Figure 3:**
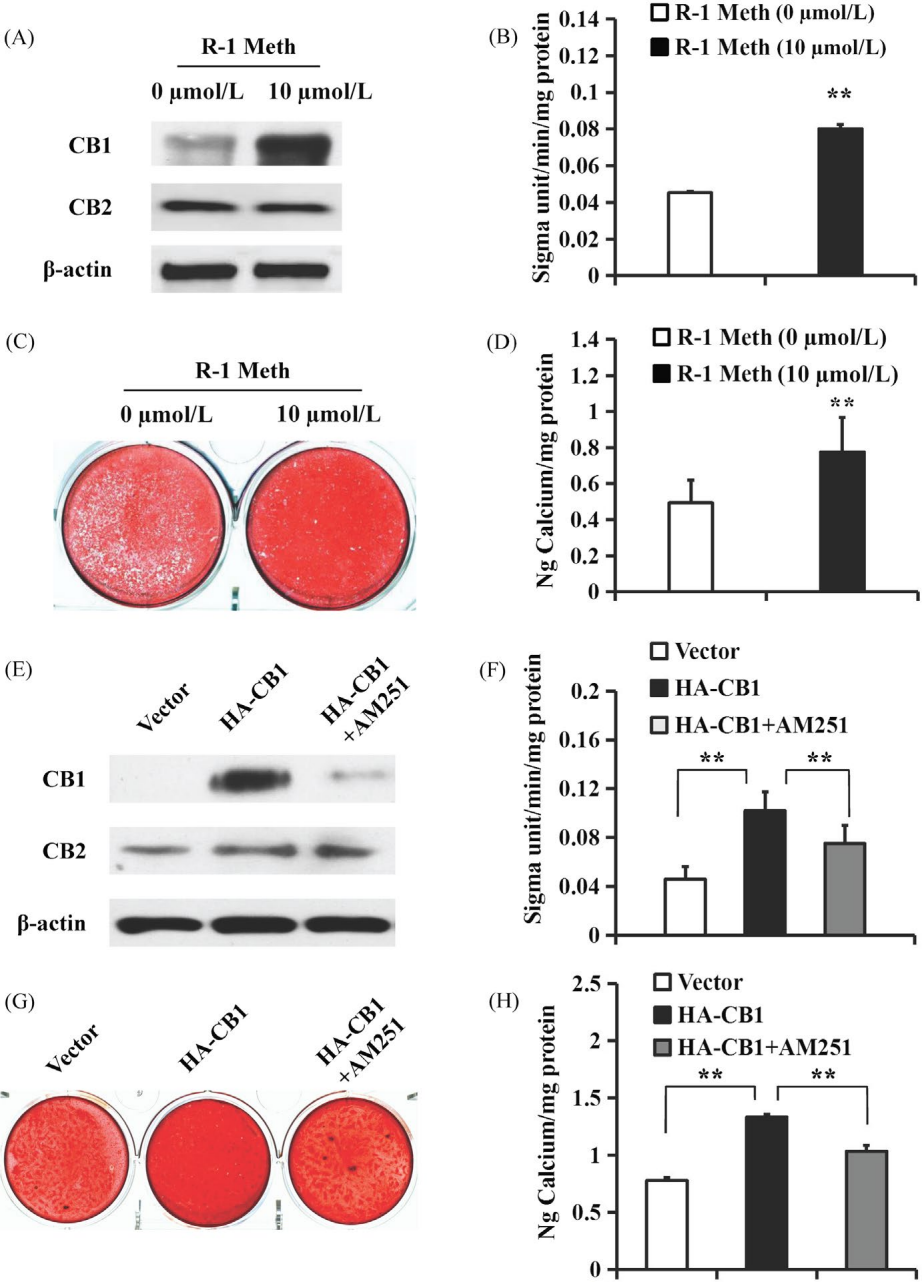
The function of R‐1 Meth and AM251 on osteo/dentinogenic differentiation in PDLSCs. A‐D, 10 µM R‐1 Meth was used to treat PDLSCs. A, Western blot results showed the expression of CB1 and CB2 in PDLSCs after treatment with 10 µM R‐1 Meth for 4 h. β‐actin was used as an internal control. B, The ALP activity assay. C, Alizarin Red staining. D, Calcium quantitative analysis. E‐H, 10 µM AM251 was used to treat the CB1‐overexpressing PDLSCs. E, Western blot results showed the expression of CB1 and CB2 in PDLSCs after treatment with 10 µM AM251 for 4 h. β‐actin was used as an internal control. F, ALP activity assay. G, Alizarin Red staining. H, Calcium quantitative analysis. Student's *t* test or one‐way ANOVA was performed to determine statistical significance. Error bars represent the SD (n = 3). **P ≤ .01

Additionally, we applied the CB1 agonist R‐1 Meth to confirm CB1 function. Western blotting showed that 10 µM R‐1 Meth activated CB1 but not CB2 (Figure 3A). After culturing in osteogenic‐inducing medium for 5 days, we found that 10 µM R‐1 Meth raised the ALP activity of PDLSCs compared with untreated PDLSCs (Figure 3B). Alizarin red staining and the quantitative calcium measurement results revealed that 10 µM R‐1 Meth promoted the in vitro mineralization of PDLSCs compared with the untreated group (Figure 3C,D).

The CB1‐specific inhibitor AM251 was then used to further confirm the role of CB1 in the osteo/dentinogenic differentiation of PDLSCs. Western blotting showed that 10 µM AM251 blocked CB1 but not CB2 (Figure 3E). After culturing the PDLSCs for 5 days in osteogenic‐inducing medium, we found that 10 µM AM251 repressed the CB1‐enhanced ALP activity of PDLSCs (Figure 3F). Alizarin red staining and the quantitative calcium measurements also revealed that 10 µM AM251 inhibited the enhanced in vitro mineralization by the overexpression of CB1 in PDLSCs (Figure 3G,H).

### 3.3 CB1 activated the p38 MAPK and JNK signals and inhibited Erk1/2 and PPAR‐γ signals in PDLSCs

**Figure 4:**
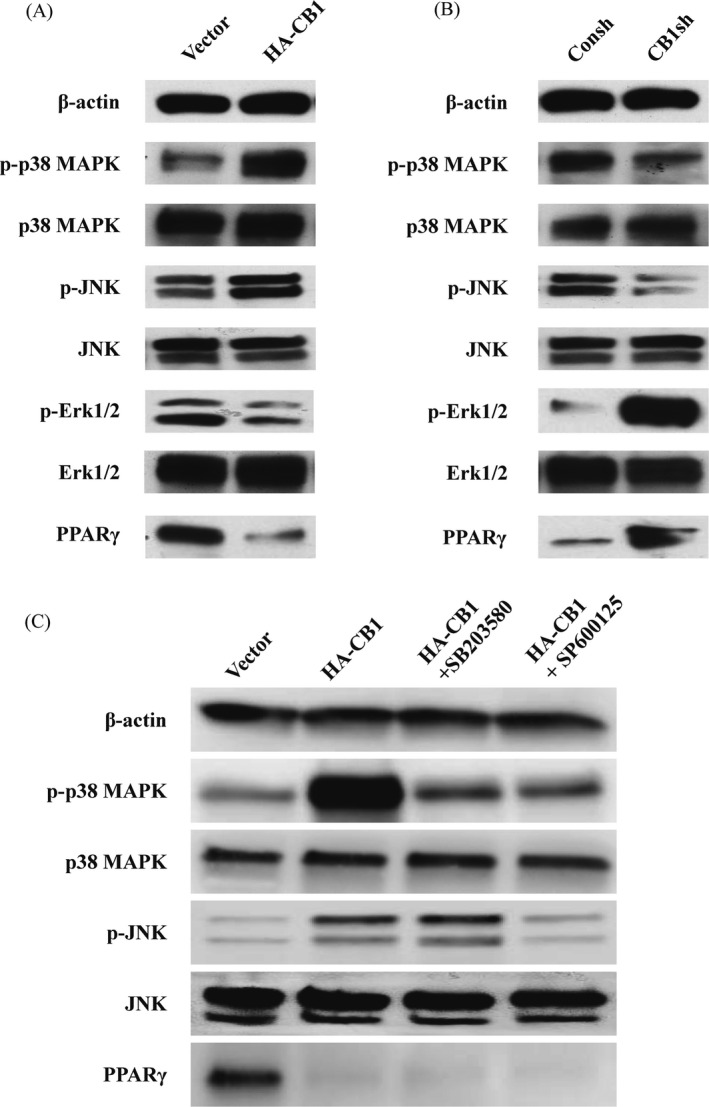
The effect of CB1 on MAPK signal pathways and PPAR‐γ in PDLSCs. A, Western blot results showed the expression of phosphorylated p38 MAPK, JNK, and Erk1/2, along with p38 MAPK, JNK, Erk1/2 and PPAR‐γ in the CB1‐overexpressing PDLSCs compared to the control group. B, Western blot results showed the expression of phosphorylated p38 MAPK, JNK and Erk1/2, along with p38 MAPK, JNK, Erk1/2 and PPAR‐γ in the CB1sh PDLSCs compared to the control group. C, 20 µM SB203580 or 20 µM SP600125 was used to treat the CB1‐overexpressing PDLSCs for 2 h. Western blot results showed the expression of phosphorylated p38 MAPK and JNK, along with p38 MAPK, JNK and PPAR‐γ in PDLSCs. β‐actin was used as an internal control

Next, Western blotting was used to ascertain the expression levels of p38 MAPK, JNK, Erk1/2 and PPAR‐γ in PDLSCs. The results showed that the levels of phosphorylated p38 MAPK and phosphorylated JNK were increased, while phosphorylated Erk1/2 and PPAR‐γ were decreased in the CB1‐overexpressing PDLSCs compared to the control group l (Figure 4A). Moreover, phosphorylated p38 MAPK and phosphorylated JNK in the CB1 knocked‐down PDLSCs were decreased, while the levels of phosphorylated Erk1/2 and PPAR‐γ were increased compared with the control group (Figure 4B).

Next, we blocked the p38 MAPK signalling pathway with its specific inhibitor, SB203580. Western blotting revealed that 20 µM SB203580 could significantly decrease the CB1‐activated phosphorylated p38 MAPK (Figure 4C). We also blocked the JNK signalling pathway with its specific inhibitor, SP600125. Interestingly, 20 µM SP600125 could significantly inhibit the CB1‐activated phosphorylated JNK and suppressed the CB1‐activated phosphorylated p38 MAPK (Figure 4C). Moreover, we found that 20 µM SB203580 and 20 µM SP600125 had no effect on PPAR‐γ expression in CB1‐overexpressing PDLSCs (Figure 4C).

### 3.4 CB1 upregulated the osteo/dentinogenic differentiation potential of PDLSCs stimulated with TNF‐α or INF‐γ

**Figure 5:**
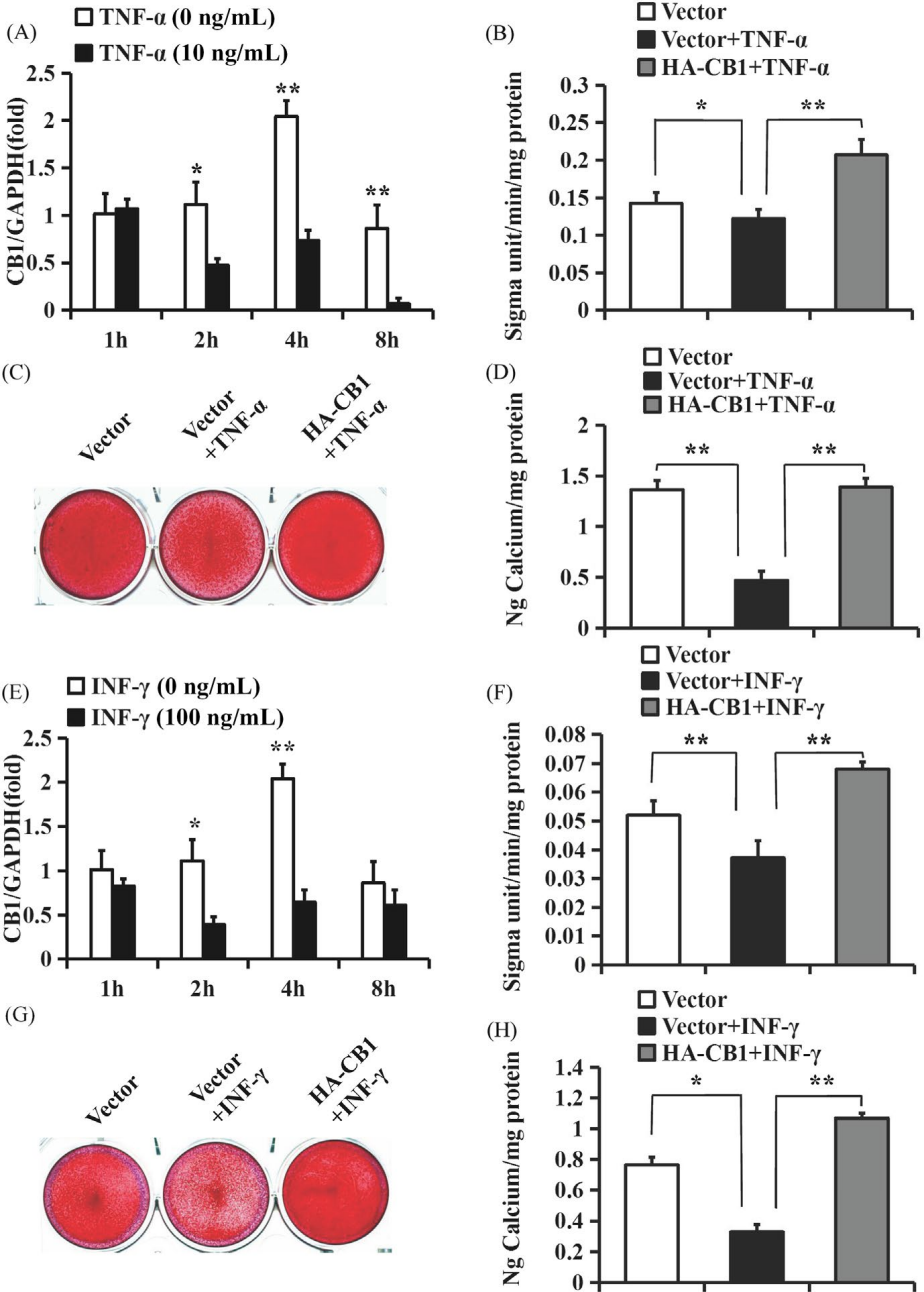
CB1 upregulated the osteo/dentinogenic differentiation potential of PDLSCs under TNF‐α and INF‐γ stimulation. A‐D, 10 ng/mL TNF‐α was used to treat PDLSCs. A, Real‐time RT‐PCR results showed the expression of CB1 at 1, 2, 4 and 8 h after 10 ng/mL TNF‐α treatment in PDLSCs. B, ALP activity assay. C, Alizarin Red staining. D, Calcium quantitative analysis. E‐H, 100 ng/mL INF‐γ was used to treat PDLSCs. E, Real‐time RT‐PCR results showed the expression of CB1 at 1, 2, 4 and 8 h after 100 ng/mL INF‐γ treatment in PDLSCs. F, ALP activity assay. G, Alizarin Red staining. H, Calcium quantitative analysis. GAPDH was used as an internal control. One‐way ANOVA was performed to determine statistical significance. Error bars represent the SD (n = 3). *P ≤ .05; **P ≤ .01

The 10 ng/mL TNF‐α or 100 ng/mL INF‐γ was used to stimulate the PDLSCs. The expression levels of IL‐6 and IL‐8 were increased at 1, 2, 4 and 8 hours after TNF‐α stimulation (Figure S1A,B), while CB1 was decreased at 2, 4 and 8 hours after 10 ng/mL TNF‐α treatment compared with the untreated group, as shown by real‐time RT‐PCR (Figure 5A). We then investigated the function of CB1 in PDLSCs upon TNF‐α treatment. The ALP activity assay results showed that 10 ng/mL TNF‐α decreased the ALP activity. Alizarin red staining and the quantitative calcium analysis results showed that 10 ng/mL TNF‐α impaired mineralization in PDLSCs, and the overexpression of CB1 could rescue this impaired ALP activity and mineralization (Figure 5B‐D). At 2 weeks after osteogenic induction, the real‐time RT‐PCR results also showed that the expressions of *ON*, *DSPP, DMP1* and *BSP* were decreased after 10 ng/mL TNF‐α treatment compared with the untreated group, and the overexpression of CB1 could rescue these gene expressions (Figure S2A‐D). Similarly, the expression of IL‐6 was increased at 1, 2 and 4 hours, IL‐8 was increased at 1, 2 and 8 hours (Figure S1C,D) and CB1 was decreased at 2 and 4 hours after 100 ng/mL INF‐γ treatment compared with untreated PDLSCs (Figure 5E). The ALP activity, Alizarin red staining and quantitative calcium measurements showed that 100 ng/mL INF‐γ decreased the ALP activity and mineralization in PDLSCs, and the overexpression of CB1 could rescue this impaired ALP activity and mineralization (Figure 5F‐H). Then, the real‐time RT‐PCR results showed that the expressions of *ON*, *DSPP, DMP1* and *BSP* were decreased after 100 ng/mL INF‐γ treatment, and the overexpression of CB1 could rescue these gene expressions (Figure S3A‐D).

**Figure 6:**
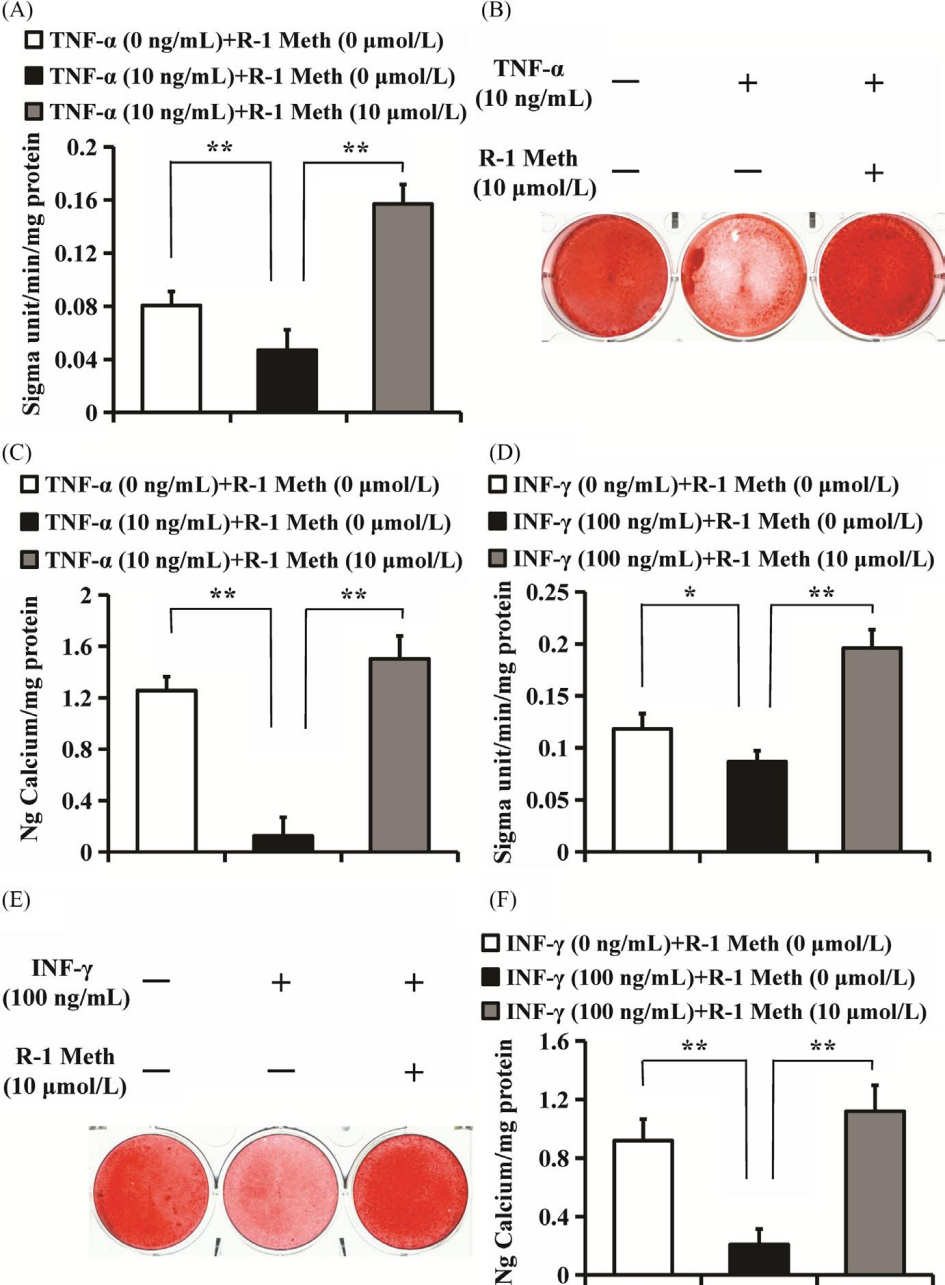
The function of R‐1 Meth on osteo/dentinogenic differentiation in PDLSCs under TNF‐α and INF‐γ stimulation. A‐C, 10 µM R‐1 Meth and 10 ng/mL TNF‐α were used to treat PDLSCs. A, ALP activity assay. B, Alizarin Red staining. C, Calcium quantitative analysis. D‐F, 10 µM R‐1 Meth and 100 ng/mL INF‐γ were used to treat PDLSCs. D, ALP activity assay. E, Alizarin Red staining. F, Calcium quantitative analysis. One‐way ANOVA was performed to determine statistical significance. Error bars represent the SD (n = 3). *P ≤ .05; **P ≤ .01

Next, we further verified the CB1 function in the osteo/dentinogenic differentiation ability of PDLSCs under inflammatory conditions by using the CB1‐specific agonist R‐1 Meth. The ALP activity assay, Alizarin red staining, quantitative calcium measurements and the real‐time RT‐PCR results indicated that 10 µM R‐1 Meth promoted the ALP activity, mineralization and the expressions of *ON*, *DSPP, DMP1* and *BSP* in PDLSCs, which was impaired under TNF‐α or INF‐γ treatment (Figure 6, Figures S4 and S5).

### 3.5 CB1 activated the p38 MAPK and JNK signals that were inhibited by TNF‐α and INF‐γ stimulation

**Figure 7:**
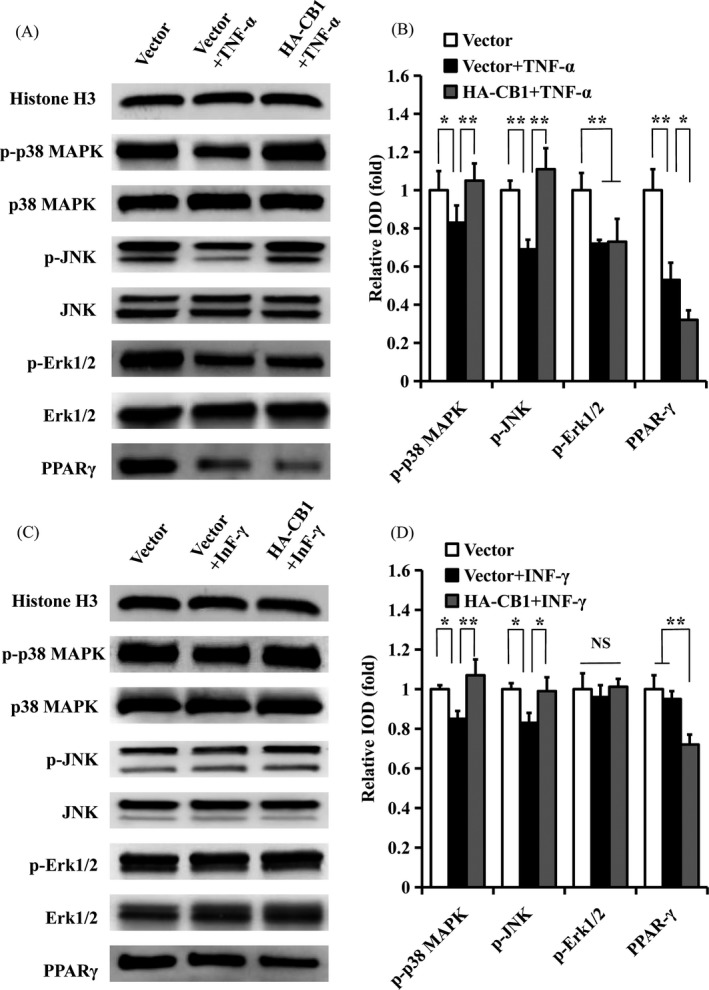
The effect of CB1 on MAPK signalling pathways and PPAR‐γ in PDLSCs under TNF‐α or INF‐γ stimulation. A, B, 10 ng/mL TNF‐α was used to treat PDLSCs for 4 h. A, Western blot showed the expression of phosphorylated p38 MAPK, JNK and Erk1/2, along with p38 MAPK, JNK, Erk1/2 and PPAR‐γ in PDLSCs. B, The quantitative analysis of phosphorylated p38 MAPK, JNK and Erk1/2 and PPAR‐γ. C, D, 100 ng/mL INF‐γ was used to treat PDLSCs for 4 h. C, Western blot showed the expression of phosphorylated p38 MAPK, JNK and Erk1/2, along with p38 MAPK, JNK, Erk1/2 and PPAR‐γ in PDLSCs. D, The quantitative analysis of phosphorylated p38 MAPK, JNK, and Erk1/2 and PPAR‐γ. Histone H3 was used as an internal control. Error bars represent the SD (n = 3). *P ≤ .05; **P ≤ .01

We further investigated the relationship among CB1, the MAPK signalling pathways and PPAR‐γ under inflammatory conditions. Western blotting revealed that the levels of phosphorylated p38 MAPK, phosphorylated JNK, phosphorylated Erk1/2 and PPAR‐γ expression were decreased in PDLSCs stimulated with 10 ng/mL TNF‐α compared with the untreated group (Figure 7A,B). The overexpression of CB1 rescued the decreased levels of phosphorylated p38 MAPK and phosphorylated JNK, and further boosted the suppressed PPAR‐γ levels that were caused by 10 ng/mL TNF‐α stimulation (Figure 7A,B). In the TNF‐α‐stimulated environment, phosphorylated Erk1/2 showed no change in the CB1‐overexpressing PDLSCs compared with the control group (Figure 7A,B). After the addition of 100 ng/mL INF‐γ, the levels of phosphorylated p38 MAPK and phosphorylated JNK were decreased, while phosphorylated Erk1/2 and PPAR‐γ expression showed no change compared with the untreated group (Figure 7C,D). The overexpression of CB1 rescued the decreased phosphorylated p38 MAPK and phosphorylated JNK levels that were caused by 100 ng/mL INF‐γ stimulation (Figure 7C,D). The overexpression of CB1 inhibited PPAR‐γ expression in the INF‐γ‐stimulated environment, but the phosphorylated Erk1/2 level showed no change (Figure 7C,D).

## 4 DISCUSSION

This study found that bacterial inflammation decreased CB1 expression in human periodontal ligament (PDL) cells.^19^ However, it has been found that CB1 seems to be upregulated during gingival wound healing in rats.^30^ To clarify the underlying role of CB1 in MSCs in mediating periodontal tissue regeneration, we explored the function and mechanism of CB1 in the osteo/dentinogenic differentiation of PDLSCs under inflammatory conditions. First, the ALP activity, in vitro mineralization, and osteo/dentinogenic markers, including *DSPP*, *DMP1*, *BSP* and *ON*, demonstrated that CB1 knock‐down decreased the osteo/dentinogenic differentiation of PDLSCs, and the overexpression of CB1 promoted osteo/dentinogenic differentiation. We then found that the osteo/dentinogenic differentiation of PDLSCs was also enhanced under the activation of CB1 by R‐1 Meth, and the CB1 inhibitor AM251 could block the CB1 overexpression‐induced osteo/dentinogenic differentiation in PDLSCs. These results indicated that CB1 could enhance the osteo/dentinogenic differentiation ability of PDLSCs. It has previously been reported that CB1 enhances the early osteogenic ability of BMSCs.^22^ Meanwhile, the deletion of CB1 causes deossification in mice.^29^ Our results are consistent with these findings. Studies have shown that several transcription factors are important during the osteo/dentinogenic differentiation of MSCs, including *RUNX2*, *OSX*, *DLX2*, *DLX3* and *DLX5*.^46^, ^47^, ^48^ Our results showed that *OSX*, *DLX2*, *DLX3* and *DLX5* expression levels were increased in CB1‐overexpressing PDLSCs and decreased in CB1‐depleted PDLSCs. However, the expression of *RUNX2* did not differ depending on the overexpression or depletion of CB1 (data not shown). These findings indicated that CB1 enhanced the osteo/dentinogenic differentiation potential of PDLSCs through upregulating *OSX*, *DLX2*, *DLX3* and *DLX5*.

Next, we investigated the role of CB1 in PDLSCs under inflammatory conditions. The development of periodontitis is tied to the accumulation of inflammatory mediators including TNF‐α and INF‐γ, and an increased inflammatory response develops with the destruction of periodontium tissue.^49^ In our study, the inflammatory factors TNF‐α and INF‐γ were used to mimic an inflammatory condition. We found that the CB1 expression level in PDLSCs was decreased after stimulation with either of these inflammatory factors. A previous study found that the inflammatory factors IL‐1β, IL‐6 and TNF‐α could enhance CB1 and CB2 expression levels in human whole blood and peripheral blood mononuclear cells (PBMCs).^50^ Upon stimulation with INF‐γ, no marked change was found in the expression level of CB1 in activated microglia.^51^ These findings indicate that inflammatory factors have different effects on CB1 in different cell types. We then found that TNF‐α and INF‐γ both inhibit the osteo/dentinogenic differentiation of PDLSCs by detecting ALP activity, in vitro mineralization, and osteo/dentinogenic markers. A previous study found that an inflammatory environment weakens the directional differentiation of PDLSCs, and it is difficult for the damaged PDLSCs to achieve the requirements of periodontal tissue regeneration.^12^ This suggests that an inflammatory environment reduces the expression of CB1 in PDLSCs, which might be associated with the impaired function of these cells. Furthermore, the overexpression of CB1 or the activation CB1 by R‐1 Meth could rescue the osteo/dentinogenic differentiation of PDLSCs that was impaired under the treatment of TNF‐α or INF‐γ. This indicates that the activation of CB1 might enhance the osteo/dentinogenic differentiation potential of PDLSCs under inflammatory conditions and could be used for enhancing PDLSC‐mediated periodontal tissue regeneration.

The primary G_i/o_‐protein‐coupled canonical endogenous ligands for the cannabinoid receptor are AEA and 2‐AG. Activation of CB1 by AEA and 2‐AG results in many biochemical responses and are frequently cell‐type specific, including the MAPK pathways.^52^ Previous research has demonstrated that CB1 activation enhances human gingival fibroblast (HGF) proliferation by activating the Erk1/2, p38 MAPK and AKT pathways.^30^ CB1 can also activate the JNK and Erk pathways in mouse osteoblasts.^53^ PPAR‐γ is primarily expressed in adipose tissue together with CB1 under normal physiological conditions; it regulates various functions such as the growth of fat cells and their ability to store lipids, and AEA can activate PPAR‐γ by CB1 receptor‐mediated signal transduction in BMSCs.^54^, ^55^ To further evaluate the regulation mechanism of CB1, we assessed the role of the MAPK signalling pathway and PPAR‐γ in CB1‐mediated regulation in PDLSCs. Our results showed that CB1 activated the p38 MAPK and JNK signalling pathways, while inhibiting the Erk1/2 signalling pathway and PPAR‐γ. Several studies have confirmed that p38 MAPK and JNK are positive regulators of osteogenic differentiation.^56^, ^57^ For example, BMP2 induces osteoblastic cell differentiation via activation of the p38 MAPK and JNK pathways.^58^ Periostin promotes the osteogenic differentiation potential of PDLSCs through the JNK pathway under inflammatory conditions.^57^ The osteogenic differentiation of PDLSCs is suppressed by IL‐7 through inactivation of the MAPK signalling pathways.^56^ IGFBP5 enhances the osteogenic differentiation potentials of MSCs via the JNK and MEK/Erk signalling pathways.^16^ Other studies have confirmed that PPAR‐γ is a negative regulator of osteogenic differentiation.^59^, ^60^ For example, a study showed that enhanced PPAR‐γ activity leads to bone loss, and reduced PPAR‐γ activity causes bone mass to increase in animal models.^59^ Moreover, high glucose levels can prevent osteogenic differentiation by activating PPAR‐γ signalling and reduces the self‐repairing ability of PDLSCs in periodontal tissues.^60^ These findings indicate that CB1 possibly enhances the osteo/dentinogenic differentiation potential of PDLSCs through activating p38 MAPK and JNK and by repressing PPAR‐γ. However, the role of Erk1/2 in the CB1‐enhanced osteo/dentinogenic differentiation potential of PDLSCs is unclear. In addition, p38 MAPK and JNK are present with a large amount of overlap in cells.^61^ PPAR‐γ activation in dendritic cells (DCs) downregulates dendritic cell‐specific ICAM‐3 grabbing non‐integrin (DC‐SIGN) expression, which is mediated by inhibiting the signalling pathways of Erk1/2 and JNK.^62^ We investigated the presence of sequences in the activation of the p38 MAPK and JNK signalling pathways on CB1 regulation and the relationship between MAPK signalling pathway and PPAR‐γ in PDLSCs. We found that when JNK signalling was blocked by its specific inhibitor (SP600125), CB1‐activated p38 MAPK signalling was inhibited in CB1‐overexpressing PDLSCs. However, the inhibition of p38 MAPK signalling by its specific inhibitor (SB203580) did not affect CB1‐activated JNK signalling. Moreover, inhibition of the p38 MAPK or JNK signalling pathways in CB1‐overexpressing PDLSCs did not affect PPAR‐γ expression. These results indicate that p38 MAPK activation relies on JNK activation by CB1, which is a downstream molecule of the JNK signalling pathway. Meanwhile, the inhibition of PPAR‐γ by CB1 is independent of the activation of p38 MAPK and JNK signalling in PDLSCs.

The MAPK signalling pathways are potential targets of inflammatory mediators and can be activated when modified by surrounding stress. Interestingly, we found that TNF‐α stimulation repressed the p38 MAPK, JNK and Erk1/2 signalling pathways. Moreover, CB1 overexpression rescued p38 MAPK and JNK signalling that was repressed under TNF‐α‐mimicked inflammatory conditions. In the TNF‐α‐stimulated environment, Erk1/2 signalling showed no change in CB1‐overexpressing PDLSCs. These results suggest that CB1 rescues the function of damaged PDLSCs via the p38 MAPK and JNK signalling pathways, but not via the Erk1/2 signalling pathway. PPAR‐γ expression was found to be decreased in PDLSCs under TNF‐α stimulation. PPAR‐γ is a negative regulator for osteo/dentinogenic differentiation, and the suppression of PPAR‐γ led to enhanced osteogenesis.^59^, ^60^ This suggests that there might be other signalling pathways, such as p38 and JNK, that compensate for the PPAR‐γ effect by TNF‐α treatment in PDLSCs. INF‐γ treatment repressed only the p38 MAPK and JNK signalling pathways in PDLSCs. However, CB1 overexpression rescued the p38 MAPK and JNK pathways that were repressed under INF‐γ‐mimicked inflammatory conditions. Under INF‐γ treatment, the overexpression of CB1 further repressed PPAR‐γ expression but did not affect the Erk1/2 signalling pathway. This suggests that CB1 promotes the osteo/dentinogenic differentiation ability of PDLSCs through p38 MAPK, JNK and PPAR‐γ in an INF‐γ‐stimulated inflammatory environment, and this process may be independent of Erk1/2. Taken together, these findings indicate that CB1 can rescue the osteo/dentinogenic differentiation ability of PDLSCs via different mechanisms under different inflammatory conditions, and the p38 MAPK and JNK signalling pathways were more important for the CB1‐activated osteo/dentinogenic differentiation potential of PDLSCs when inhibited by inflammation.

## 5 CONCLUSION

In conclusion, our findings revealed that CB1 could activate the osteo/dentinogenic differentiation potential of PDLSCs under inflammatory conditions. This effect might be mediated via activation of the p38 MAPK and JNK signalling pathways and the repression of PPAR‐γ. In addition, our results suggest that the CB1‐activated p38 MAPK signalling pathway is dependent on JNK signalling, and inhibition of PPAR‐γ by CB1 is independent of p38 MAPK and JNK signalling. More importantly, our discoveries suggest that p38 MAPK and JNK are key molecules for the CB1‐activated osteo/dentinogenic differentiation potentials of PDLSCs when they are impaired by inflammation. Our results clarified the potential role and mechanism of CB1 in PDLSCs under inflammatory conditions and provide candidate targets for enhancing MSC function and the treatment of periodontitis.

## CONFLICTS OF INTEREST

The authors deny any conflicts of interest related to this research.

## Data Availability

Research data are not shared.
