# Supplementary material for: CB1 enhanced the osteo/dentinogenic differentiation ability of periodontal ligament stem cells via p38 MAPK and JNK in an inflammatory environment
Source: Cell Prolif. 2019 Oct 10;52(6):e12691. doi: 10.1111/cpr.12691 (PMC6869632; doi:10.1111/cpr.12691)
Supplement: Supplementary file 1 [file CPR-52-e12691-s001.doc]

**SUPPLEMENTARY MATERIAL**

**Supplementary table 1. The primers for specific genes used in Real-time RT-PCR**

| **Specific Genes** | **Target Sequences** |
| --- | --- |
| GAPDH-Forward  GAPDH-Reverse  OSX-Forward  OSX-Reverse  ON-Forward  ON-Reverse  BSP-Forward  BSP-Reverse  DSPP-Forward  DSPP-Reverse  DMP1-Forward  DMP1-Reverse  DLX2-Forward  DLX2-Reverse  DLX3-Forward  DLX3-Reverse  DLX5-Forward  DLX5-Reverse  CB1-Forward  CB1-Reverse  IL-6-Forward  IL-6-Reverse  IL-8-Forward  IL-8-Reverse | 5’‑CGGACCAATACGACCAAATCCG-3’ 5’‑AGCCACATCGCTCAGACACC-3’ 5’‑CCTCCTCAGCTCACCTTCTC-3’ 5’‑GTTGGGAGCCCAAATAGAAA-3’ 5’‑TCCCTGTACACTGGCAGTTC-3’ 5’‑TTGTCCAGGTCACAGGTCTC-3’ 5’‑CAGGCCACGATATTATCTTTACA-3’ 5’‑CTCCTCTTCTTCCTCCTCCTC-3’ 5’‑CGACATAGGTCACAATGAGGATGTCG-3’ 5’‑TTGCTTCCAGCTACTTGAGGTC-3’ 5’‑CGTGGACAAAGAAGATAGCAACTCCACG-3’ 5’‑TTCCGGCTCTCTATCTCAATGTTT-3’ 5’‑ATGCACTCGACCCAGATCG-3’ 5’‑GGCTTGGTACTGGTAGGAACC-3’ 5’‑TACCCTGCCCGAGTCTTCTG-3’  5’‑TGGTGGTAGGTGTAGGGGTTC-3’ 5’‑TTCCAAGCTCCGTTCCAGAC-3’ 5’‑GAATCGGTAGCTGAAGACTCG-3’ 5’-CGGACCAATACGACCAAATCCG-3’ 5’-AGCCACATCGCTCAGACACC-3’  5’-CGCAACAACTCATCTCATTCTGCG-3’  5’-CATGCTACATTTGCCGAAGAGC-3’  5’-CGGATAAAGGGCCAAGAGAATATCCG-3’  5’-TCACATTCTAGCAAACCCATTCAA-3’ |


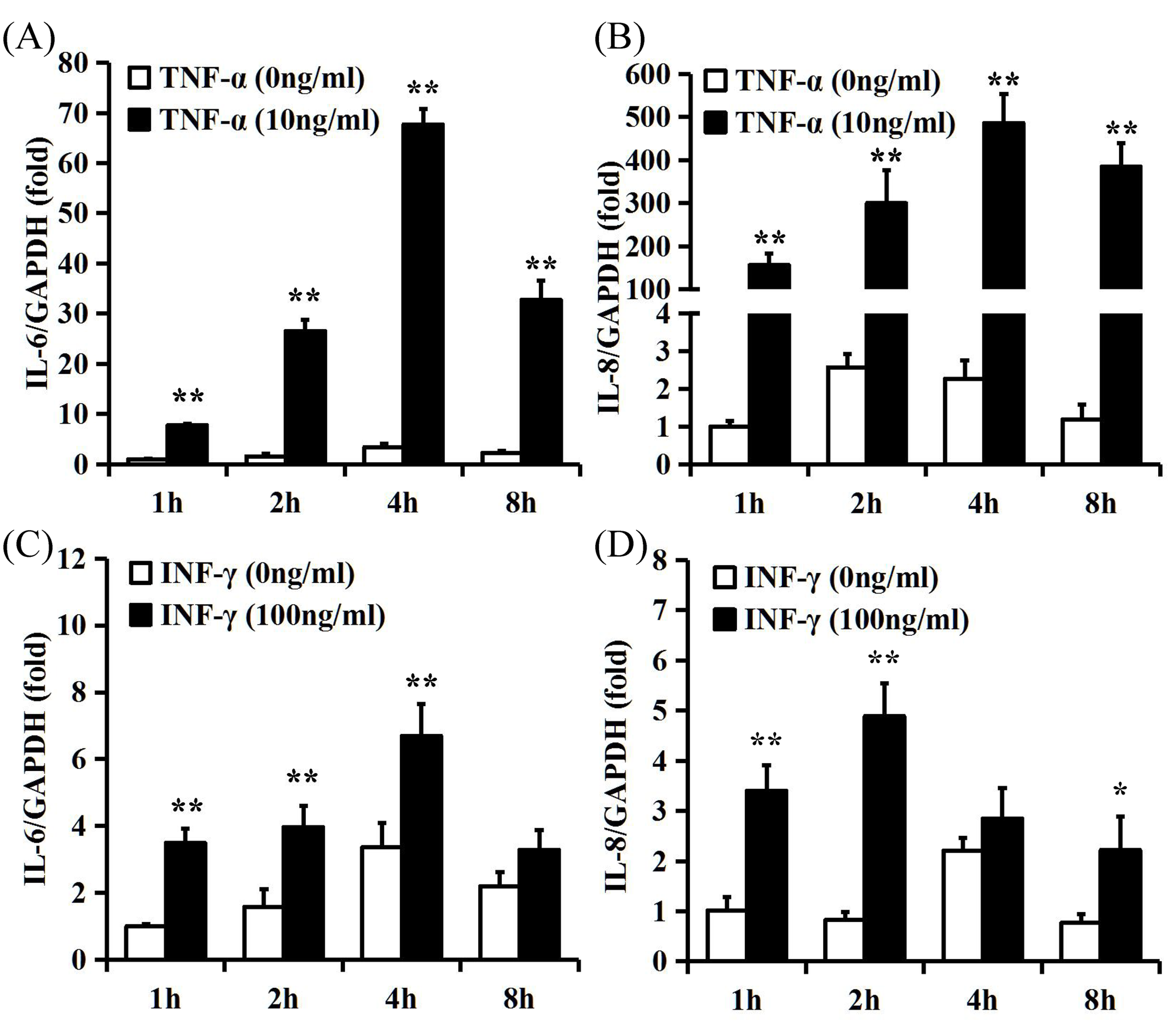


**Supplementary Figure 1. The expressions of IL-6 and IL-8 in PDLSCs after TNF-α or INF-γ stimulation.** (A, B) Real-time RT-PCR results showed the expression of IL-6 (A) and IL-8 (B) at 1, 2, 4 and 8 h after 10 ng/ml TNF-α treatment in PDLSCs. (C, D) Real-time RT-PCR results showed the expression of IL-6 (C) and IL-8 (D) at 1, 2, 4 and 8 h after 100 ng/ml INF-γ treatment in PDLSCs. GAPDH was used as an internal control. Student’s t-test was performed to determine statistical significance. Error bars represent the SD (n= 3). *P ≤ 0.05; **P ≤ 0.01.


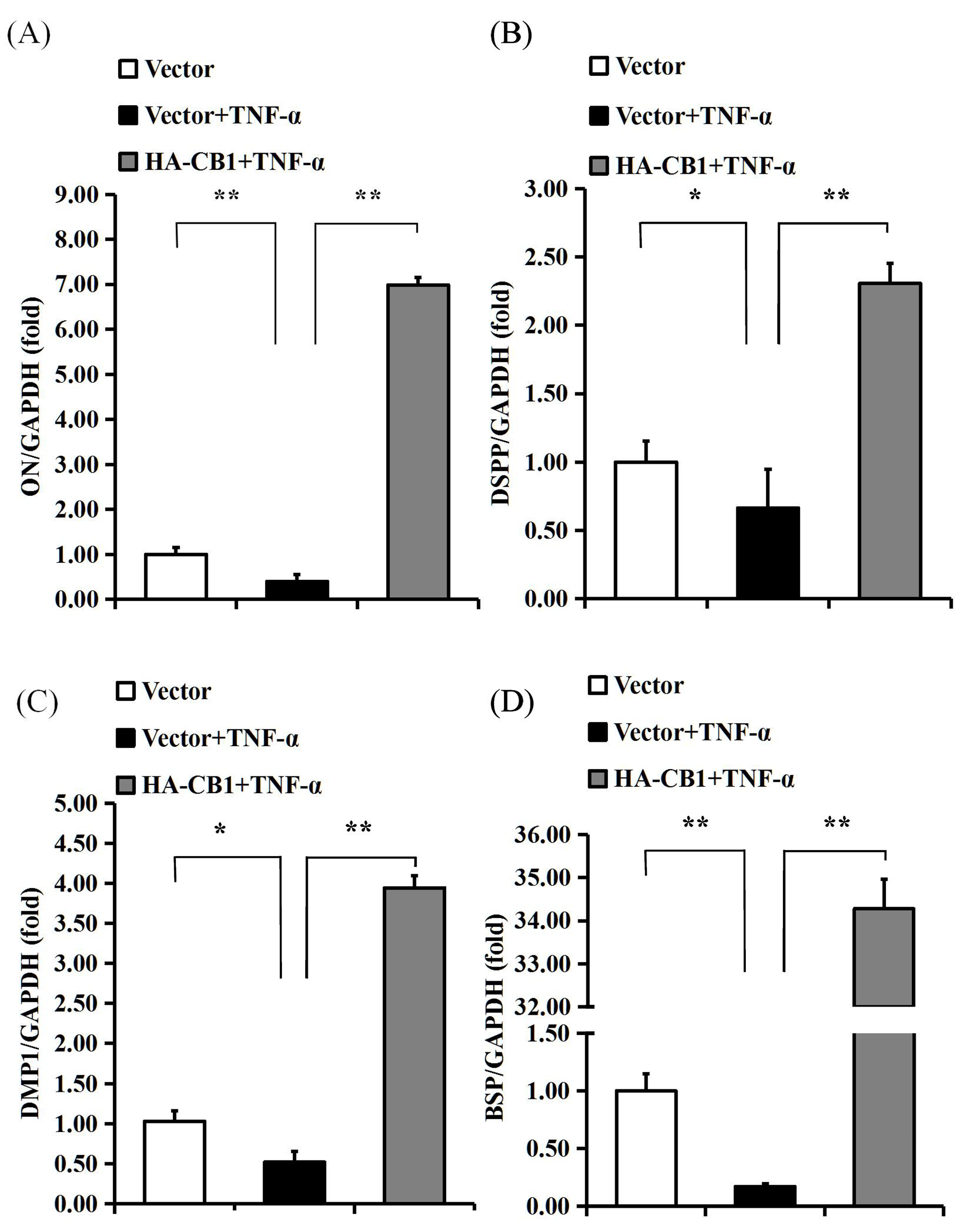


**Supplementary Figure 2. CB1 upregulated the expressions of osteo/dentinogenic differentiation markers in PDLSCs under TNF-α stimulation.** 10 ng/ml TNF-α was used to treat PDLSCs. (A-D) At 2 weeks after osteogenic induction, the Real-time RT-PCR results showed the expressions of *ON* (A), *DSPP* (B), *DMP1* (C), and *BSP* (D) in PDLSC after 10 ng/ml TNF-α treatment. GAPDH was used as an internal control. One-way ANOVA was performed to determine statistical significance. Error bars represent the SD (n= 3). *P ≤ 0.05; **P ≤ 0.01.


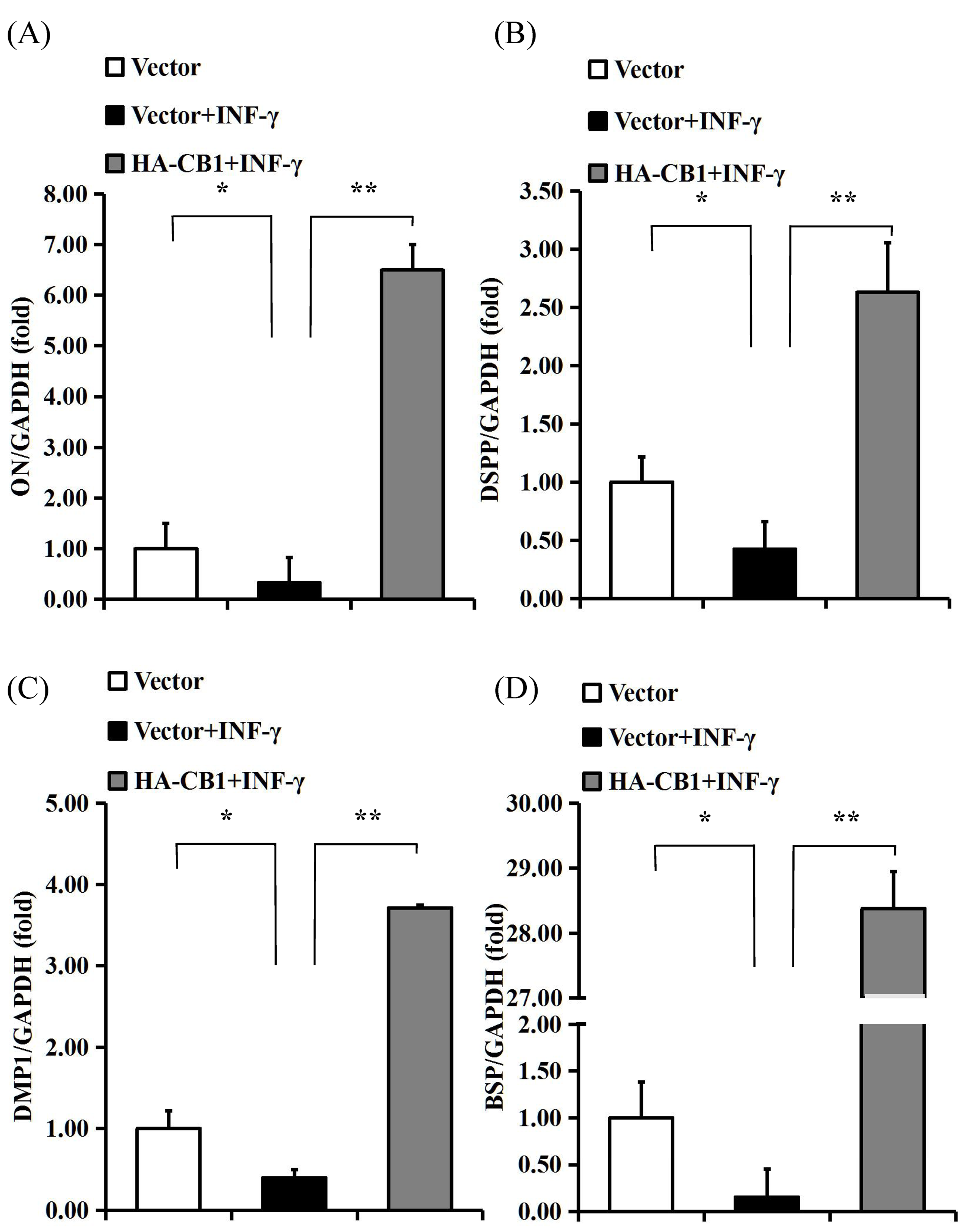


**Supplementary Figure 3. CB1 upregulated the expressions of osteo/dentinogenic differentiation markers in PDLSCs under INF-γ stimulation.** 100 ng/ml INF-γ was used to treat PDLSCs. (A-D) At 2 weeks after osteogenic induction, the Real-time RT-PCR results showed the expressions of *ON* (A), *DSPP* (B), *DMP1* (C), and *BSP* (D) in PDLSC after 100 ng/ml INF-γ treatment. GAPDH was used as an internal control. One-way ANOVA was performed to determine statistical significance. Error bars represent the SD (n= 3). *P ≤ 0.05; **P ≤ 0.01.


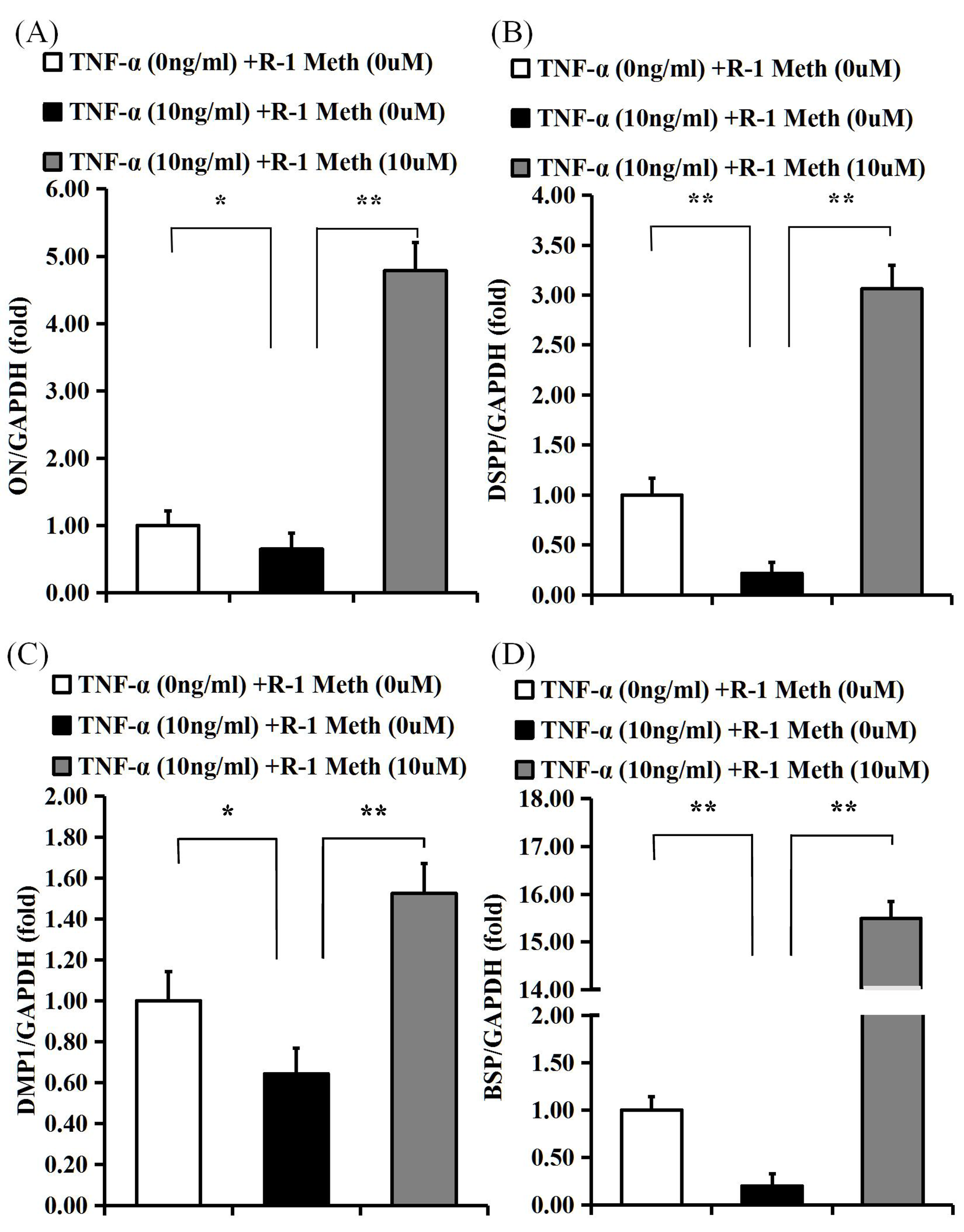


**Supplementary Figure 4. The function of R-1 Meth on the expressions of osteo/dentinogenic differentiation markers in PDLSCs under TNF-α stimulation.** 10 ng/ml TNF-α was used to treat PDLSCs. (A-D) At 2 weeks after osteogenic induction, the Real-time RT-PCR results showed the expressions of *ON* (A), *DSPP* (B), *DMP1* (C), and *BSP* (D) in PDLSC after 10 ng/ml TNF-α treatment. GAPDH was used as an internal control. One-way ANOVA was performed to determine statistical significance. Error bars represent the SD (n= 3). *P ≤ 0.05; **P ≤ 0.01.


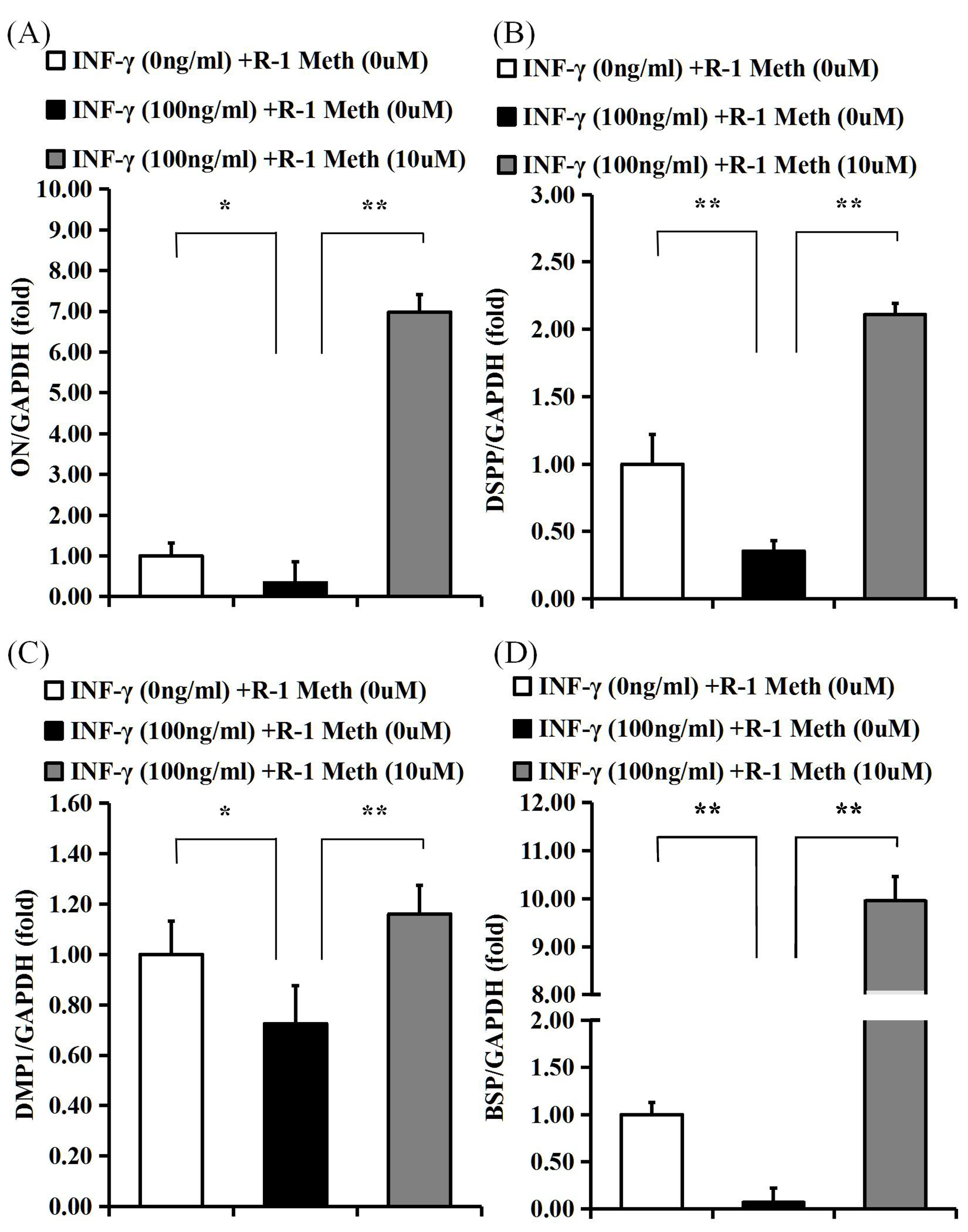


**Supplementary Figure 5. The function of R-1 Meth on the expressions of osteo/dentinogenic differentiation markers in PDLSCs under INF-γ stimulation.** 100 ng/ml INF-γ was used to treat PDLSCs. (A-D) At 2 weeks after osteogenic induction, the Real-time RT-PCR results showed the expressions of *ON* (A), *DSPP* (B), *DMP1* (C), and *BSP* (D) in PDLSC after 100 ng/ml INF-γ treatment. GAPDH was used as an internal control. One-way ANOVA was performed to determine statistical significance. Error bars represent the SD (n= 3). *P ≤ 0.05; **P ≤ 0.01.
